# Process Study on 3D Printing of Polymethyl Methacrylate Microfluidic Chips for Chemical Engineering

**DOI:** 10.3390/mi16040385

**Published:** 2025-03-28

**Authors:** Zengliang Hu, Minghai Li, Xiaohui Jia

**Affiliations:** 1 School of Mechanical Engineering, Dalian Jiaotong University, Dalian 116028, China; dlminghai@vip.sina.com; 2 School of Chemical Engineering and Machinery, Liaodong University, Dandong 118001, China; x1170329696@163.com

**Keywords:** 3D-printing technology, polymethyl methacrylate substrate, microchannel roughness, orthogonal experiment method, finite element method, chemical engineering, microfluidic technology

## Abstract

Microfluidic technology is an emerging interdisciplinary field that uses micropipes to handle or manipulate tiny fluids in chemistry, fluid physics, and biomedical engineering. As one of the rapid prototyping methods, the three-dimensional (3D) printing technique, which is rapid and cost-effective and has integrated molding characteristics, has become an important manufacturing technology for microfluidic chips. Polymethyl-methacrylate (PMMA), as an exceptional thermoplastic material, has found widespread application in the field of microfluidics. This paper presents a comprehensive process study on the fabrication of fused deposition modeling (FDM) 3D-printed PMMA microfluidic chips (chips), encompassing finite element numerical analysis studies, orthogonal process parameter optimization experiments, and the application of 3D-printed integrated microfluidic reactors in the reaction between copper ions and ammonium hydroxide. In this work, a thermal stress finite element model shows that the printing platform temperature was a significant printing parameter to prevent warping and delamination in the 3D printing process. A single printing molding technique is employed to fabricate microfluidic chips with square cross-sectional dimensions reduced to 200 μm, and the microchannels exhibited no clogging or leakage. The orthogonal experimental method of 3D-printed PMMA microchannels was carried out, and the optimized printing parameter resulted in a reduction in the microchannel profile to Ra 1.077 μm. Finally, a set of chemical reaction experiments of copper ions and ammonium hydroxide are performed in a 3D-printed microreactor. Furthermore, a color data graph of copper hydroxide is obtained. This study provides a cheap and high-quality research method for future research in water quality detection and chemical engineering.

## 1. Introduction

Microfluidic chips (chips) represent a dynamic and continually advancing area of research that offers numerous advantages to both chemical and biological investigations. These include minimizing the quantity of samples and reagents required, shortening experimental durations, and facilitating the creation of in vivo-like conditions [1]. The current technologies for fabricating microfluidic chips mainly include the LIGA technique [2], photolithography [3], laser ablation [4], and 3D printing [5], among others. At present, three-dimensional (3D) printing technology, also known as additive manufacturing technology, has the advantage of rapid prototyping to reduce costs and has become a crucial technique for rapidly prototyping microfluidic devices [6]. Various 3D-printed technologies have been explored for the fabrication of microfluidic devices, including stereolithography (SLA) [7], inkjet printing [8], multi-jet printing [9], and fused deposition modeling (FDM) [10]. Khoo et al. proposed a rapid prototyping protocol to fabricate thermoplastic devices from an SLA 3D printing template through intermediate steps akin to those employed in soft lithography. This process was applied to the design of self-operating capillary circuits and is well suited for deployment in low-cost decentralized assays [11].

Compared to whole 3D printing processing methods, fused deposition modeling (FDM) technology, with faster prototyping, shorter lead times for manufacturing, and lower production costs, has gained considerable attention. Fused deposition modeling (FDM) is an additive manufacturing technology commonly used for modeling, prototyping, and production applications. FDM works using an additive principle by laying down material in layers, and a plastic filament is unwound from a coil and supplies material to produce a part. PMMA, also known as acrylic glass or acrylic, is a transparent, lightweight, and durable thermoplastic with excellent optical clarity and weather resistance [12]. Its application in microfluidic systems offers numerous advantages, including ease of fabrication, biocompatibility, and the ability to create complex, three-dimensional structures. Furthermore, the use of PMMA in microfluidic systems has been shown to provide excellent optical clarity, making it suitable for imaging and analysis applications.

Scientific research has demonstrated the feasibility and potential of 3D printing PMMA microfluidic chips. Urrios et al.’s study showed that PMMA-based microfluidic devices could be used for cell culture applications, with cells growing well within the devices [13]. Bressan et al. presented a solvent bonding method of polymethyl methacrylate and acrylonitrile butadiene styrene (ABS) thermoplastic materials for the creation of optically detectable 3D-printed microfluidic devices [14]. Kotz et al. demonstrated that using FDM, microfluidic chips with a minimum channel cross-section of ~300 µm can be printed and a variety of different channel geometries and mixer structures are shown. Researchers further demonstrate that protein patterns can be generated within previously printed microfluidic chips by employing a method of photobleaching [15]. Thana et al. demonstrated that 3D printers with three-dimensional (3D) printing, computer numerical control (CNC) milling, and laser engraving technology were an effective strategy for creating microfluidic devices and an easier and more economical alternative to, for instance, conventional photolithography [16]. Plastic microfluidic chips with engraved microchannel structures or micro-structured plastic molds for casting polydimethylsiloxane (PDMS) chips with microchannel imprints were produced. Akbari et al. discussed the microfabrication approaches used for creating inertial microchannels, including photolithography, xerography, laser cutting, micromachining, microwire technique, etching, hot embossing, 3D printing, and injection molding [17]. The advantages and disadvantages of these methods were also discussed. Tsao et al. gave a short overview of polymer microfabrication methods for microfluidics and discussed current challenges and future opportunities for research in polymer microfluidics fabrication [18]. Gyimah et al. evaluated the potential of the large-scale application of 3D-printed microfluidics [19]. Cutuli et al. proposed a multi-objective polydimethylsiloxane (PDMS) micro-optofluidic (MoF) device suitably designed and manufactured through a 3D-printed-based master–slave approach [20].

In conclusion, FDM 3D-printed technology with a layer-by-layer extrusion mechanism resulted in channel resolutions typically >200 μm, which struggled to meet the sub-200 μm requirements for advanced microfluidics applications. Surface roughness, caused by filament stacking, led to fluid leakage or inefficient flow control. While the FDM PMMA microfluidic chip faced challenges in resolution and material performance, its low cost and adaptability made it indispensable for research and niche applications. Through systematic optimization of FDM 3D-printed parameters, this work developed a low-cost fabrication process for PMMA microfluidic chips capable of producing 200 μm-scale channels. The optimized chips exhibited enhanced mechanical stability, reduced thermal deformation, and reliable fluidic performance, achieved by effectively mitigating clogging and leakage risks.

This paper presents the design and fabrication of FDM 3D-printed PMMA microfluidic devices for chemical reaction engineering. The FDM 3D-printed parameters are optimized through iterative experiments, and a single-step printing molding technique is developed. This integrated approach simplifies the fabrication workflow by eliminating the need for post-printing bonding processes. Firstly, numerical simulation is integrated with experimental trials to analyze thermal stress evolution during FDM 3D-printed PMMA, enabling the identification of critical printing parameters that mitigate warping and delamination through optimized thermomechanical control. Secondly, an L9(3^3^) orthogonal experimental design is implemented to systematically optimize printing parameters, resulting in a significant reduction in the fabricated microchannel surface roughness. Thirdly, the microreactor structure is designed and fabricated via a single-step integrated 3D-printed process, utilizing optimized parameters to achieve monolithic construction with enhanced structural integrity. Finally, a set of chemical reactions between copper ions and ammonium hydroxide are conducted, resulting in a blue-colored copper hydroxide solution.

## 2. Materials and Methods

### 2.1. Three-Dimensional Printing Materials

In the experiment, PMMA as an organic polymer material was studied. The polymer substrate possessed characteristics of high transparency, excellent toughness and very good dimensional stability. The density of PMMA was 1.18 g/cm^3^, and the temperature of glass transition is 104 °C. The transmittance of PMMA was about 92% in infrared spectral range. PMMA was well-suited for 3D printing owing to its thermoplastic properties. The material was melted and extruded via fused deposition modeling (FDM) systems, enabling the fabrication of high-precision microfluidic architectures with intricate geometries. In this experiment, PMMA filament was purchased from Chinese shopping website.

### 2.2. FDM 3D Printing System

**Figure 1:**
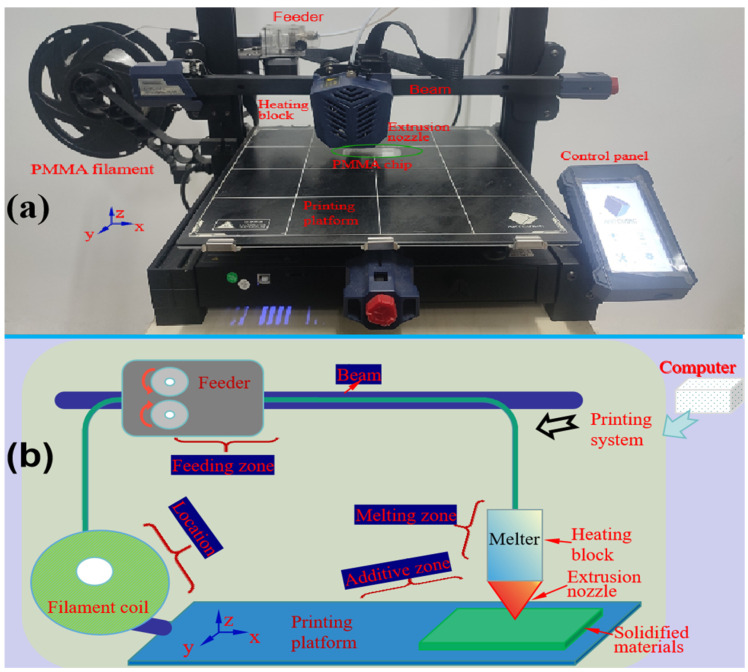
The structure and schematic diagram of the FDM 3D-printed process. (**a**) The structure diagram of FDM 3D-printed process. (**b**) The schematic diagram of FDM 3D-printed process.

During the 3D printer printing the target parts, all designs were created using SolidWorks design software 2022 and exported as STL files in computer. In this research, the STL files were imported into JGcreat 4.8.6 for the slicing process. The microchannel structure was designed in SolidWorks software v2022 and then imported into slicing software for processing. The relevant machining parameters were set in the slicing software, which can automatically complete the trajectory data extraction, trajectory planning, simulation, and interference check. The FDM printer Anycubic Kobra Plus (Anycubic Technology, Shenzhen, China) was used for printing. Figure 1a shows the structure diagram of FDM 3D-printed process.

Figure 1b shows the schematic diagram of FDM 3D-printed process. The 3D printer consisted of a location zone, a feeding zone, a melting zone and an additive manufacturing zone. A beam was mounted on a stabilizing frame that allowed movement along the *x-*, *y-*, and *z*-axes. The positioning zone served to initially position the filament material, ensuring that it could enter the feeding zone accurately and smoothly. The feeding zone consisted of a drive gear and an idler pulley with an integrated bearing, where a precisely calibrated gap was maintained between the two components to ensure controlled filament traction. The melting zone consisted of an extrusion nozzle and a heating block with thermal insulation layer. Within the heating block, filaments were melted in a pressurized chamber via a piston-driven mechanism, which extrudes the molten material through the nozzle. The additive manufacturing zone included workbench and workpiece. The additive manufacturing process was performed on workbench, where molten filament was extruded from nozzle. The melt zone was mounted on a three-axis gantry system, which precisely controlled movement along the *x-*, *y-*, and *z*-axes. The *z*-direction determined the thickness of each printed layer. On the 3D printer, the control panel enabled functions to suspend, start, and stop the printing process.

### 2.3. Finite Element Analysis

During the preliminary phase of process development, FDM 3D printing of PMMA filament was simulated using COMSOL Multiphysics software v5.5, a finite element analysis software platform for multiphysics computational modeling. Heat transfer in solids interface was used to simulate heat transfer by conduction and convection. A solid model was active by default on the simulation domains. The temperature equation defined in the solid domains was governed by the differential form of Fourier’s law. There were two equations as calculative tools in the simulation. 

The simulation was explained by the following Fourier Heat equations:

(1) $$\rho C_{p}\frac{\partial T}{\partial t}+\rho C_{p}u\cdot \nabla T+\nabla \cdot (-k\nabla T)=Q+Q_{ted}$$

where *ρ* represents density, *C_p_* stands for specific heat capacity, *T* denotes temperature, *K* is the thermal conductivity coefficient, *Q* signifies heat flux, and *Q_ted_* is the thermoelastic effect.

In order to obtain a stable thermodynamic model, a point heat source was set with the equation.

(2) $$\theta =\rho c\left(x^{2},+,y^{2},+,z^{2}\right)$$

where *ρ* is the density of the heat-conducting medium and *c* is the specific heat capacity of the heat-conducting medium.

This time-dependent study was supported in three dimensions, and PMMA material was used for the simulation process. To reduce computational load while ensuring accuracy, this simulation focused on a single-layer model featuring a cuboid of 0.1 mm and divided the finite element mesh into ultra-fine mesh elements.

**Figure 2:**
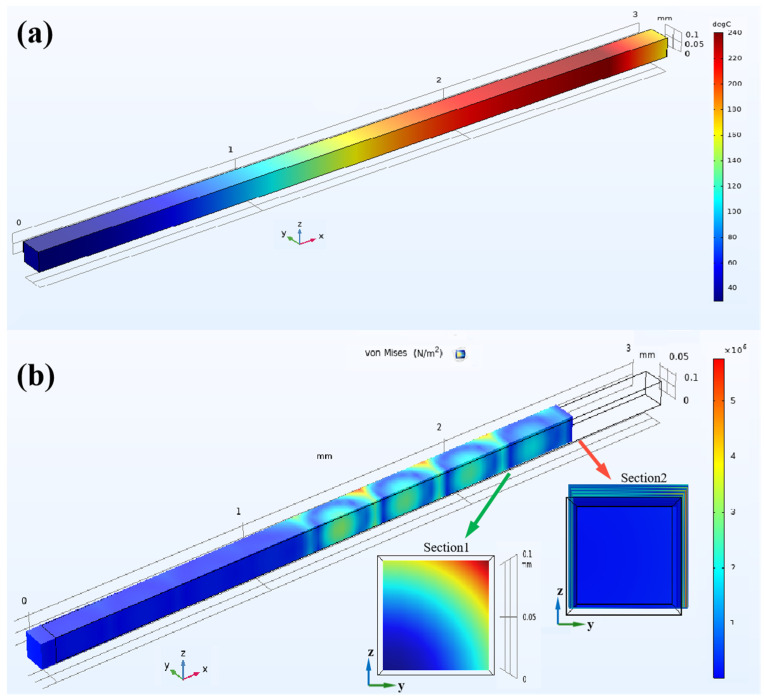
The simulation diagram of the temperature situation and thermal stress distribution with the 3D-printed PMMA process. (**a**) Temperature situation. (**b**) Thermal stress distribution.

Figure 2 reflects the actual temperature and thermal stress distribution in the FDM 3D-printed PMMA process. The actual temperature distribution on the PMMA printed surface at 0.6 s is shown in Figure 2a. In the diagram, the range from 0 mm to 3 mm represented the printing direction of the material. The high-temperature area moved along this printing direction, and the already printed sections rapidly cooled down to room temperature as printing progressed. During the layer-by-layer additive manufacturing process, heat accumulation occurred due to conductive thermal transfer between material layers, while non-uniform temperature gradients and differential thermal expansion induced residual stresses within the deposited structure.

Due to heat transfer, thermal stress arose on the surface of the printing layer, as illustrated in Figure 2b. The color legend on the right indicated the distribution of the Von Mises stress, which arose from thermal conduction and heat accumulation during the additive manufacturing process. The thermal stress distribution analysis revealed the progressive evolution of internal stresses during the additive manufacturing process, with particular emphasis on scenarios characterized by minimal substrate adhesion. Temporal analysis revealed that residual thermal stresses in the initially deposited layer were observed to progressively diminish, while a tripartite stress concentration configuration developed within newly deposited layers during subsequent manufacturing phases. The green arrow pointed to the side view Section 1, which not only delineated the directional stress gradient characteristics in the *zy*-plane but also revealed the vector alignment between principal thermal stress concentrations and the resultant thermo-mechanical load vector. According to the cross-sectional analysis of the stress gradient, a gradual decrease in magnitude was observed from the upper right corner to the lower left corner of the *zy*-plane, with the maximum stress concentration located in the upper right region. The red arrow highlighted Section 2 in the side view aligned with the *x*-axis. In this section, the black outline corresponded to the designed contour, whereas the numerically simulated contour deviated toward the *zy*-plane, suggesting potential thermal–structural coupling effects.

### 2.4. Discussion of 3D Printing Parameter

**Figure 3:**
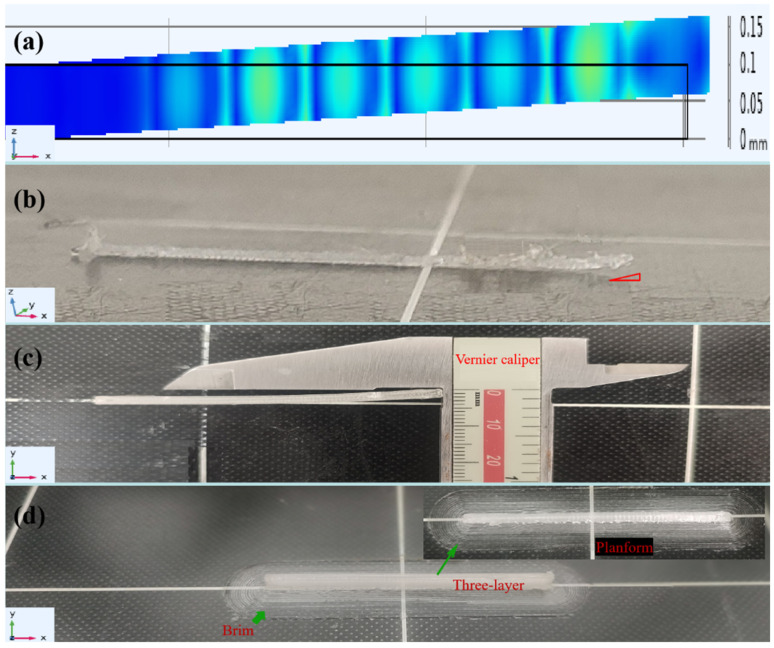
The warping diagram material additive with the FDM 3D-printed PMMA process. (**a**) Printing material warping deformation with the numerical simulation system. (**b**) Printing material warping deformation with an actual machining system. The red triangle represents the degree of horizontal warping of the printed single-layer material relative to the platform. (**c**) The PMMA material warps with three layers printing along the vertical route in the *Y*-axis direction. (**d**) The morphology of optimally printed three-layer PMMA materials, warping and delamination suppression via platform temperature control (95 °C), and brim adhesion strategy: synergistic effects in 3D-printed PMMA microfluidic chip fabrication.

Figure 3 presents a comparative analysis of warping deformation along the *xz*-axis in PMMA printed materials between experimental measurements and numerical simulations at matched spatial coordinates. The corresponding numerical simulations and experimental measurements under identical printing parameters are presented in Figure 3a and Figure 3b, respectively. Both figures exhibited similar *z*-axis warping trends in the PMMA material, with deformation increasing progressively during printing. Figure 3c illustrates the *y*-axis warping deformation of PMMA material after three-layer deposition along a vertical build path. A vernier caliper with precision markings was used to quantify the maximum warping displacement as 0.2 mm, measured relative to the reference horizontal line on the printing platform. The observed deformation direction aligned quantitatively with the simulated thermal stress gradient vector field (Figure 2b, Section 1, green arrow), confirming the thermomechanical coupling effects during printing.

To mitigate warping and interlayer delamination, a single-factor experimental design was implemented to systematically identify critical process parameters. Figure 3d shows the defect-free microstructure of the PMMA specimen achieved through the optimized printing strategy. Horizontal alignment of the PMMA structure along the bed's reference line was achieved through precise temperature control of the build platform. The experimental results showed the printing effect of PMMA material was optimal without warping and delamination issues when the printing platform temperature was set to 95 °C. Furthermore, the printing platform attachment type was also one of the key factors to enhance the stability of printed objects. Implementing a brim attachment type around the model base improved mechanical stability and reduced thermal contraction stresses. These discoveries were crucial for ensuring the accuracy and integrity of the printed parts.

**Figure 4:**
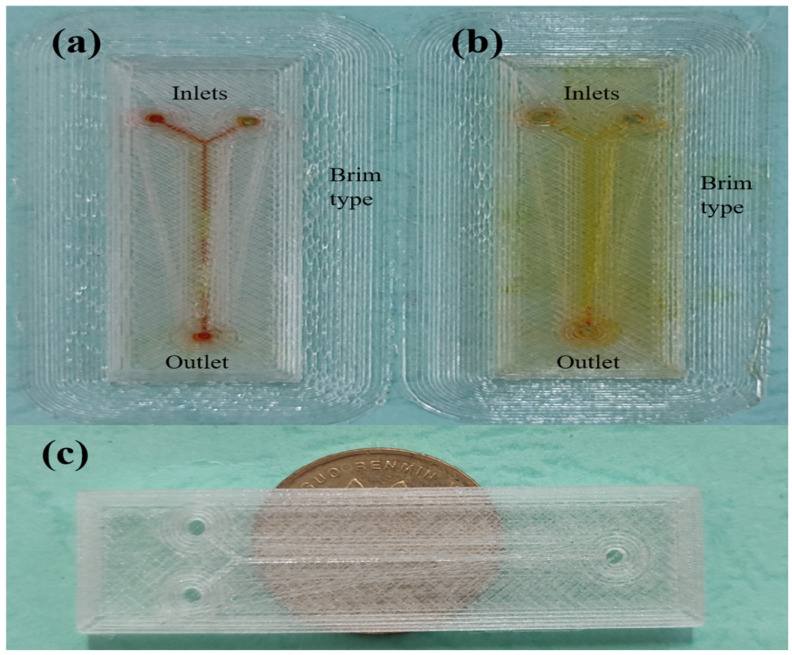
The profile of a microfluidic chip with a single 3D-printed molding technique. (**a**) Leakage-free 3D-printed microfluidic chips enabled by gyroid infill pattern: structural integrity enhancement through TPMS design. (**b**) Fluid leakage in 3D-printed chips with twelve other infill patterns (e.g., rectilinear, triangular, and hexagonal): structural deficiencies from reduced integrity and mechanical strength. (**c**) Optical clarity and structural homogeneity of 2-mm-thick 3D-printed PMMA microfluidic chips: transparency benchmarking against a microreactor and a 50-cent CNY coin for scale reference.

Figure 4 shows side leakage and transparency test results for samples produced through a single printing molding technique with different processing parameters. A single printing molding technique bypassed traditional chip bonding and streamlined fabrication processes. The sample microfluidic chip had dimensions of 50 mm in length, 2 mm in thickness, and 15 mm in width in this test. The cross-section of the designed microchannel was a square with a side length of 0.3 mm. This work ultimately revealed that while the infill pattern was a crucial factor in preventing leakage from the sealed chip during printing, other parameters, including nozzle extrusion temperature, layer height, and print platform temperature, also contributed to maintaining structural integrity. The gyroid, a triply periodic minimal surface (TPMS) structure, exhibited outstanding physical properties. Compared to other twelve structural patterns (e.g., triangular, square, and hexagonal), the structure could significantly enhance strength and increase specific surface area. Red or yellow ink was employed in this experiment owing to their high visual contrast under optical monitoring. Figure 4a depicts the state of the chip without leakage, and Figure 4b shows the state of the chip with leakage. During the printing process, an underlying layer was added for enhancing chip stability. Figure 4c shows the optical transparency analysis of a microfluidic chip sample with a thickness of 2 mm alongside its surface morphological characterization. This was a comparison photograph between the microreactor and a fifty-cent yuan coin, which clearly showed the transparency of the 3D-printed PMMA microfluidic chip.

**Figure 5:**
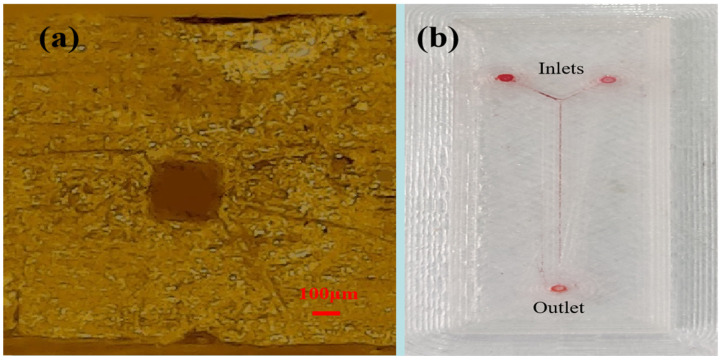
The profile of a microfluidic chip with a microchannel of 200 μm. (**a**) The shape of microchannel cross-sections of 200 μm with a metallographic microscope. (**b**) The microchannel of 200 μm without leak and clog.

Based on the above discussion of experimental parameters and PMMA material properties, the parameters of the FDM 3D-printed process were as follows: a layer height of 0.1 mm, an infill of 100%, a printing speed of 30 mm/s, a nozzle temperature of 220 °C, a fill pattern of gyroid, a printing platform attachment type of brim, and a platform temperature of 95 °C. Printing parameter optimization enabled the fabrication of PMMA microchannels via 3D printing, achieving a minimum channel width of 200 μm. The thickness of the microfluidic chip was set to 1.5 mm to ensure clearer transparency. The channel cross-section with the size of 200 μm was a similar rectangle-shaped design, as shown in Figure 5a. This minor deviation was observed and was attributed to the phase transition phenomenon involving melting and re-solidification of the materials. The printing material underwent shrinkage during the solidification process, which led to a dimension change of microchannels. At the micrometer scale, even minor changes of material flowability could result in discrepancies of cross-section between the design and the printed microchannel. Printing of channels with both width and height below 200 μm resulted in partial clogging and was therefore not further investigated. Figure 5b shows that it did not leak and clog. Red ink was employed in this experiment owing to its high visual contrast under optical monitoring.

### 2.5. Roughness Analysis of Orthogonal Method

During FDM 3D printing of a PMMA microfluidic chip, anisotropic thermal expansion coefficients of PMMA during heating, extrusion, and cooling processes induced molecular chain orientation and cooling rate gradients. These factors led to non-uniform material shrinkage, causing dimensional deviation and blockage of microchannel cross-section. Additionally, the instantaneous temperature difference between the molten PMMA layer (upper) and the solidified layer (lower) during sequential deposition could reach 120 °C, generating an inhomogeneous thermal stress field. The rapid cooling-induced shrinkage of the upper layer imposed tensile stress on the underlying layers, resulting in interlayer delamination or microcrack formation, which compromised the mechanical stability of the chip. The thermo-mechanical coupling behavior during 3D printing PMMA was critical for improving microfluidic chip precision. Addressing these challenges requires an optimized processing parameter to balance manufacturing efficiency with functional reliability of the device.

**Figure 6:**
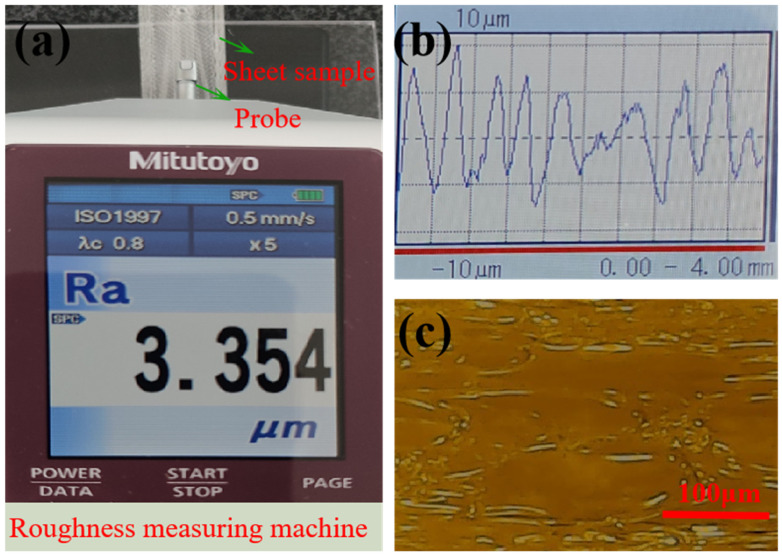
The roughness survey diagram of a microchannel with a FDM 3D-printed process. (**a**) Roughness value. (**b**) Roughness waves. (**c**) The micrograph of the microchannel profile using metallographic microscopy (scale bar: 100 μm).

The impact of microchannel surface roughness on a microfluidic chip was multifaceted, encompassing flow speed, flow state, reaction efficiency, mixing effect, chip performance and stability, as well as detection results and accuracy. Therefore, during the design and fabrication of a microfluidic chip, it was necessary to strictly control the surface roughness to ensure that the performance and stability of the chip meet the practical application requirements. A set of experiments on the surface roughness of microchannels using a single 3D-printed technique were performed. In order to ensure the accuracy of measuring data, the printing PMMA microchannels were cleaned by the ultrasonic cleaning machine. After achieving the cleaning microchannel, ten measurement points were selected in each microchannel. The data of roughness were obtained by the roughness measuring machine with a probe. Figure 6 shows the experimental system of measuring roughness and the micrograph of the PMMA microchannel profile. The roughness measuring machine selected is the SJ-210 of the Mitotoyo Company (Kawasaki City, Kanagawa Prefecture, Japan), and the testing standard is the international roughness measurement standard (ISO1997) [21]. Figure 6a,b show the roughness value and roughness waviness of the microchannel profile using the roughness measuring machine. The roughness value (*Ra*) was 3.354 μm. Due to the instability of the experiment, the experimental data have great volatility. The final data were the average of ten datasets. Figure 6c shows the microchannel profile was not regular using metallographic microscopy.

For convenience, the optimal conditions were obtained by a few experiments using the orthogonal experiment method. The nozzle temperature, printing speed, and layer height are defined as the main factors to influence the microchannel profile in the experiment. A L9(3^3^) 3D-printed orthogonal array experiment using orthogonal array experimental factors was performed. The orthogonal levels and factors were listed in Table 1. Table 2 showed the corresponding parameter of the experiment of each group and the factors response.

The average values of ten sets of microchannel roughness was obtained through microscopic testing and are summarized in Table 2. The processing stability was defined as follows:

(3) $$H=\frac{\sum_{i}^{m}S_{i}-S_{i\max}-S_{i\min}}{\left(m-2\right)}$$

where S_imax_ was the maximum roughness of the microchannel profile and S_imin_ was the minimum roughness of the microchannel.

*T*_1_, *T*_2_, and *T*_3_ respectively indicated the average of factors in each level. The computational formula was defined as follows:

(4) $$T_{i}=\frac{\sum H_{i}}{3}$$

where *i* is the number of levels and *l* is the number of factors.

*R* represented the sensitivity of the various factors and was calculated by Equation (5).

(5) $$R=\left|T_{\max}-T_{\min}\right|$$

where *T_max_* and *T_min_* showed maximum and minimum in the whole array, respectively.

In Table 2, range *R* represented the influencing results of all factors. The influence of maximum and minimum was nozzle temperature and layer height among all parameters, respectively.

**Figure 7:**
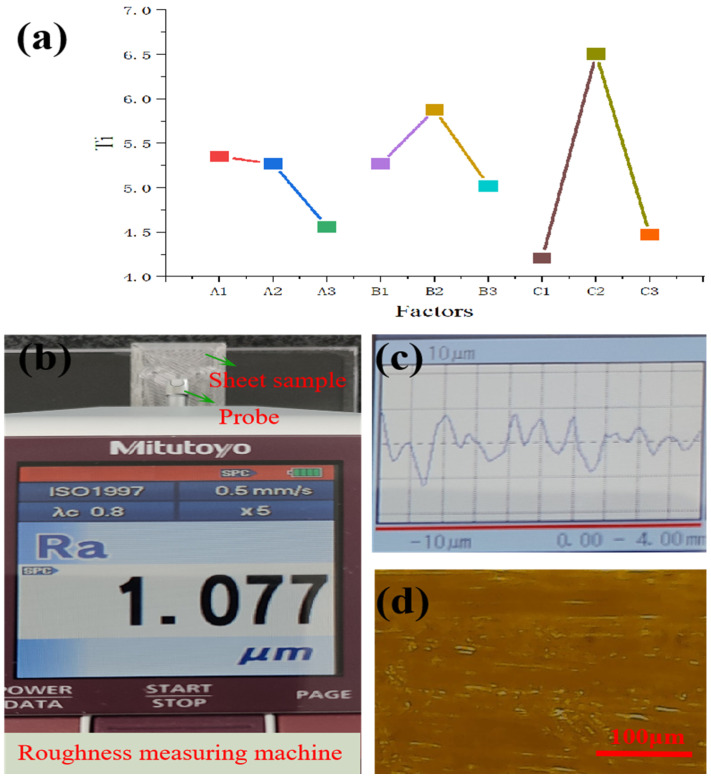
The optimized result of PMMA microchannel with an orthogonal method. (**a**) A factor response figure of the orthogonal method. *Ai* was the factor of nozzle temperature, *Bi* was the factor of printing speed, and *Ci* was the factor of layer height. The line was steeper; the influence of the factor was greater. (**b**) Roughness value with optimized 3D-printed parameter. (**c**) Microchannel profile waviness with optimized 3D-printed parameter. The waviness characterized the microchannel surface planarity. (**d**) The micrograph of the optimized microchannel profile using metallographic microscopy (scale bar: 100 μm).

In order to clearly express the effect of factors in Table 2, a factor response figure was presented. Figure 7a shows a factor response figure. In the figure, the line was steeper, and the influence of the factor was greater. The line representing layer height was close to the vertical, so it was the most sensitive factor for processing improvement. The level combination of better 3D-printed parameters in the experiment was A3B3C1. The layer height was 0.1 mm, the printing speed was 35 mm/s, and the nozzle temperature was 220 °C. According to the result, Figure 7b and Figure 7c, respectively, present the surface roughness value and waviness of PMMA microchannel fabricated with optimized 3D printing parameters in the measuring system. The roughness of the microchannel profile was 1.077 μm using the roughness measuring machine. Figure 7d shows that the micrograph of the microchannel profile with optimized 3D-printed parameters was regular and smooth using metallographic microscopy.

## 3. Results

### 3.1. Microreactor Design

As one of the heavy metal ions, copper ions played multiple roles in nature and modern science and technology applications. An adequate amount of copper ion could participate in the composition and activation of various enzymes, which was crucial for maintaining normal physiological functions. However, when the concentration of copper ions exceeded the normal range in the body, it could exert toxic effects. Excessive copper ions accumulated in organs such as the liver and kidneys, leading to dysfunction or even failure of these organs. Excessive intake of copper ions in water was one of the main causes of copper poisoning, underscoring the critical need for real-time detection of these ions. Microfluidic chips, characterized by real-time integrated detection, were one of the important ways to detect heavy metals in water. This work designed a set of experiments for copper ion detection utilizing a chemical reaction-based colorimetric method.

The reaction between copper ions and a small amount of ammonium hydroxide (commonly known as ammonia water) resulted in the formation of a blue precipitate of copper(II) hydroxide. It is typically represented in the following chemical equation form:

(6) $$Cu^{2+}+2NH^{3}\cdot H_{2}O=Cu{\left(OH\right)}^{2}+2NH^{4+}$$

**Figure 8:**
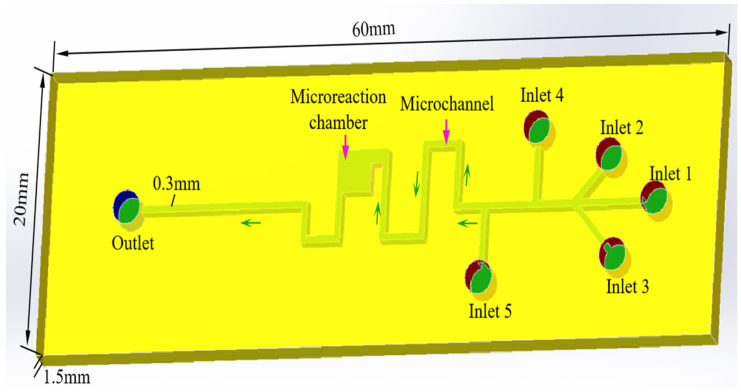
The three-dimensional structure diagram of the microreactor.

To successfully carry out this experiment, a microreactor on a microfluidic chip with five inlets and one outlet was designed using SolidWorks 2022 software. Due to the low molar mass injected in the sample, it was difficult for the reactants to develop color within the microchannel. Therefore, the five inlets were incorporated into the design to facilitate the injection of varying volumes of solutions. The sizes of the microreactor were a depth of 2 mm, a width of 20 mm, and a length of 60 mm. This microchannel size was the rectangular shape of 0.2 mm, and a reaction chamber with a length and width of 3 mm each was designed in the microchannel for fully realizing the reaction effect. The direction of the green arrow was the direction of the fluid flow. The structure diagrams of the microreactor are shown in Figure 8. The microchannel featuring a square-wave pattern was designed to improve reaction efficiency. In addition, all structures were left–right symmetrical in this study.

### 3.2. Copper-Ion Detection Experiment

In microreactor systems, the coupling effects between reaction kinetics and hydrodynamic behavior were pivotal in determining reaction efficiency and product homogeneity. The low-Reynolds-number flow regime within the designed microchannels ensured that the mixing of copper ions and ammonia was predominantly governed by laminar interfacial diffusion rather than turbulent agitation. When the hydrodynamic residence time significantly exceeded the reaction characteristic time, the reaction approached near-complete conversion. To balance reaction yield and throughput requirements, this condition allowed for the optimization of hydrodynamic residence time by tuning the flow rates. In this study, the serpentine channel geometry and flow rate parameter jointly dictated the fluid residence time distribution. By strategically engineering velocity fields within the microchannels, high-shear zones could be generated at laminar interfaces, establishing a shear-mediated regulatory mechanism for particle aggregation behavior. These high-shear regions suppressed Cu(OH)_2_ (copper hydroxide) deposition by intensifying particle fragmentation, thereby mitigating channel clogging risk while maintaining reaction continuity.

**Figure 9:**
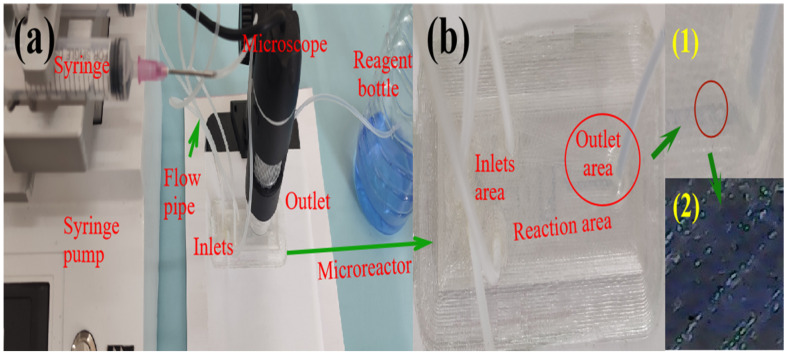
Experimental process diagram of microreactor. (**a**) Experimental system diagram of chemical reaction with copper ion and ammonium hydroxide in microreactor. (**b**) Overall morphology of microreactor. (1) Local appearance in the output area. (2) Magnified picture of copper hydroxide at the output area by microscope.

In this experiment, the rubber hoses were used to connect syringes and inlet holes of the microreactor. The copper ions and ammonium hydroxide were purchased from a Chinese shopping website. The standard values of these two solutions were 1000 μg/mL and 10%, respectively. The Reynolds number was a key parameter for distinguishing between laminar and turbulent flow in fluids, so adjusting the flow rate of the syringe pump was the most important operation in this study. In addition, the flow rate of the syringe pump was obtained from multiplying the flow velocity by the cross-sectional area of the microchannel. As shown in Figure 9a, all experimental settings for copper ion detection were presented. The varying shades of blue in the microreactor outlet were observed by stereoscopic microscope.

Figure 9b shows the chemical reaction process of copper ion and ammonium hydroxide in a microreactor. The copper ion and ammonium hydroxide were simultaneously injected into the microchannel through the hose at the same speed. In Figure 9b, the overall morphology of the microreactor, including the inlet area, reaction area, and output area, is represented. Figure 9(b1) displays the local view of the outlet area, clearly demonstrating the presence of a blue copper hydroxide solution. Figure 9(b2) shows a magnified view of the blue copper hydroxide precipitate, captured using a microscope.

### 3.3. Discussion of the Experiment

**Figure 10:**
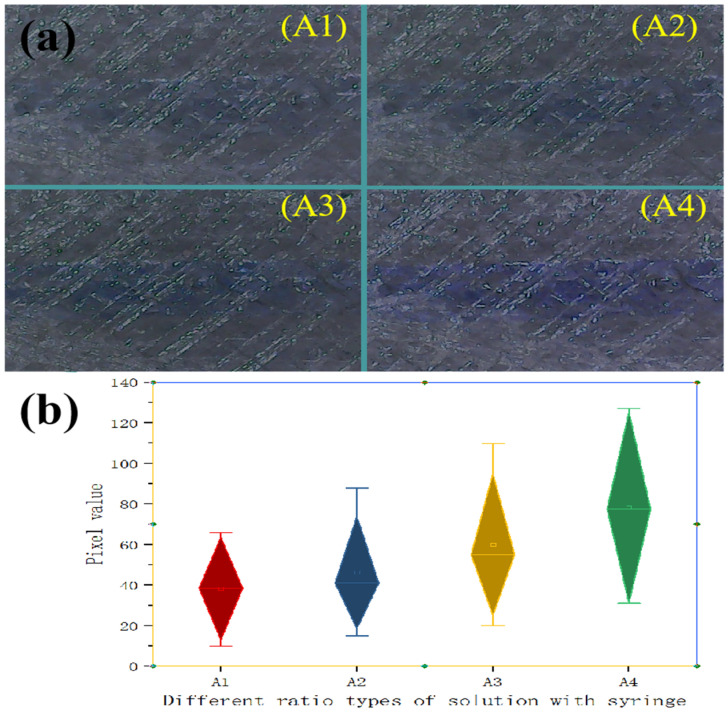
The color diagram and color pixel data of copper hydroxide with different numbers of copper ions and ammonium hydroxide. (**a**) The color diagram with different numbers of copper ions and ammonium hydroxide. (**A1**) The copper hydroxide color diagram with one syringe containing copper ions and five syringes containing ammonium hydroxide. (**A2**) The copper hydroxide color diagram with two syringes containing copper ions and four syringes containing ammonium hydroxide. (**A3**) The copper hydroxide color diagram with three syringes containing copper ions and two syringes containing ammonium hydroxide. (**A4**) The copper hydroxide color diagram with four syringes containing copper ions and one syringe containing ammonium hydroxide. (**b**) The color pixel data of copper hydroxide with different numbers of copper ions and ammonium hydroxide corresponding to A1, A2, A3, and A4.

To better observe the reaction dynamics between varying concentrations of copper ions and ammonia in a microchannel, a series of experimental tests was conducted. This experiment was implemented by altering the substances in five syringes. The color intensity variations of copper hydroxide, formed by solutions containing different concentrations of copper ions, are shown in Figure 10a. Figure 10a(A1–A4) shows the color variation of copper hydroxide corresponding to the use of one, two, three, and four syringes containing copper ion solutions, respectively. The gradual intensification of the blue coloration in Figure 10a(A1–A4) correlated with higher amounts of reactants. This suggested that the proportion of copper ions in the microchannel was a key factor influencing the intensity of the copper hydroxide color. Furthermore, it became challenging to discern the coloration of copper hydroxide when it was injected into the microchannel in proportionally small quantities.

To better analyze the experimental results, the pixel values of the blue channel in each dataset were calculated and plotted as shown in Figure 10b. The horizontal axis of the box plot was labeled as one, two, three, and four syringes containing copper ions, corresponding to the experimental groups. The figure clearly showed a linear relationship between the blue channel pixel value and the number of syringes containing copper ions. By employing a box plot, the reaction between various copper ions and ammonium hydroxide could be numerically analyzed. This method supported the integration of microreaction chips with mobile devices to enhance data acquisition in subsequent studies.

## 4. Conclusions

A 3D-printing technique with the advantages of low cost, rapid prototyping, and the creation of complex structures has garnered significant attention for the manufacture of microfluidic devices. In this work, the processing technology of PMMA microfluidic chips using a commercial FDM 3D printer was investigated, and the chemical experiment was performed in a 3D-printed PMMA microreactor.

The finite element numerical analysis method was used to predict the change of thermal stress in the process of 3D-printed PMMA material. The results of numerical modeling showed that the printing platform temperature was one of the critical printing parameters for preventing warping and delamination in the experiment. The experimental results showed that the printing performance of the PMMA material was optimal, with no warping or delamination, when the platform temperature was set to 95 °C and the attachment type was configured as brim. This design effectively prevented the PMMA materials from moving or deforming during printing, thereby improving both print accuracy and success rate. During fabrication, a single-step 3D-printing technique was used to produce microfluidic chips exhibiting high optical clarity, with embedded channels featuring square cross-sections of 200 μm. The microchannel exhibited no clogging or leakage. Through orthogonal experiments, it was revealed that nozzle temperature is an important factor affecting the microchannel profile roughness. Optimized 3D-printed parameters were obtained to reduce the microchannel profile roughness to Ra1.077 μm.

Excessive copper ions in water or food posed health risks to humans. A chemical reaction system based on microfluidic chips for copper ions and ammonium hydroxide has been designed and prepared using optimized 3D-printed parameters. To obtain copper hydroxide solutions with varying shades of blue, the microreactor was equipped with five inlets, each corresponding to a syringe. All syringes contained the same molar mass of sample solution. By adjusting the combination of ammonium hydroxide and copper ion solutions in five syringes, varying shades of blue in the copper hydroxide product were observed at the outlet. Ultimately, a box color data graph of copper hydroxide was presented. This work provided a convenient, inexpensive, and rapid method for future heavy metal detection in water. Future works will focus on integrating 3D-printed water quality detection chips with smartphones and promoting high-quality development of microfluidic chips.

## Figures and Tables

**Table 1:** Orthogonal levels and factors.

Levels	Factor		
	A (Nozzle Temperature) (°C)	B (Printing Speed) (mm/s)	C (Layer Height) (mm)
1	210	25	0.1
2	220	30	0.15
3	230	35	0.2

**Table 2:** The factor responses.

No.	A	B	C	Average Value H (μm)
1	1	1	1	4.111
2	1	2	2	7.988
3	1	3	3	3.964
4	2	1	2	6.158
5	2	2	3	3.915
6	2	3	1	5.736
7	3	1	3	5.544
8	3	2	1	2.776
9	3	3	2	5.374
T1	5.354	5.271	4.208	
T2	5.270	5.880	6.507	
T3	4.565	5.025	4.474	
R	0.79	0.85	2.30	

## Data Availability

The data are available from the corresponding author upon reasonable request.
